# Identifying congenital generalized lipodystrophy using deep learning-DEEPLIPO

**DOI:** 10.1038/s41598-023-27987-5

**Published:** 2023-02-07

**Authors:** Natália Bitar da Cunha Olegario, Joel Sotero da Cunha Neto, Paulo Cirillo Souza Barbosa, Plácido Rogério Pinheiro, Pedro Lino Azevêdo Landim, Ana Paula Dias Rangel Montenegro, Virginia Oliveira Fernandes, Victor Hugo Costa de Albuquerque, João Batista Furlan Duarte, Grayce Ellen da Cruz Paiva Lima, Renan Magalhães Montenegro Junior

**Affiliations:** 1 Brazilian Group for the Study of Inherited and Acquired Lipodystrophies (BRAZLIPO), Clinical Research Unit, Walter Cantidio University Hospital, Federal University of Ceará/EBSERH, Rua Coronel Nunes de Melo 1142, Fortaleza, Ceara 60416-000 Brazil; 2 Center of Technology, University of Fortaleza, Fortaleza, Brazil; 3 Postgraduate Program in Applied Informatics, University of Fortaleza, Fortaleza, Brazil; 4 Department of Teleinformatics Engineering, Federal University of Ceará, Fortaleza, Brazil; 5 Department of Clinical Medicine, Federal University of Ceará, Fortaleza, Brazil; 6 Postgraduate Program in Public Health, Federal University of Ceará, Fortaleza, Brazil

**Keywords:** Diseases, Endocrinology, Health care, Signs and symptoms, Engineering

## Abstract

Congenital Generalized Lipodystrophy (CGL) is a rare autosomal recessive disease characterized by near complete absence of functional adipose tissue from birth. CGL diagnosis can be based on clinical data including acromegaloid features, acanthosis nigricans, reduction of total body fat, muscular hypertrophy, and protrusion of the umbilical scar. The identification and knowledge of CGL by the health care professionals is crucial once it is associated with severe and precocious cardiometabolic complications and poor outcome. Image processing by deep learning algorithms have been implemented in medicine and the application into routine clinical practice is feasible. Therefore, the aim of this study was to identify congenital generalized lipodystrophy phenotype using deep learning. A deep learning approach model using convolutional neural network was presented as a detailed experiment with evaluation steps undertaken to test the effectiveness. These experiments were based on CGL patient’s photography database. The dataset consists of two main categories (training and testing) and three subcategories containing photos of patients with CGL, individuals with malnutrition and eutrophic individuals with athletic build. A total of 337 images of individuals of different ages, children and adults were carefully chosen from internet open access database and photographic records of stored images of medical records of a reference center for inherited lipodystrophies. For validation, the dataset was partitioned into four parts, keeping the same proportion of the three subcategories in each part. The fourfold cross-validation technique was applied, using 75% (3 parts) of the data as training and 25% (1 part) as a test. Following the technique, four tests were performed, changing the parts that were used as training and testing until each part was used exactly once as validation data. As a result, a mean accuracy, sensitivity, and specificity were obtained with values of [90.85 ± 2.20%], [90.63 ± 3.53%] and [91.41 ± 1.10%], respectively. In conclusion, this study presented for the first time a deep learning model able to identify congenital generalized lipodystrophy phenotype with excellent accuracy, sensitivity and specificity, possibly being a strategic tool for detecting this disease.

## Introduction

Congenital generalized lipodystrophy (CGL) is a rare autosomal recessive disease characterized by near complete absence of functional adipose tissue from birth. That reduction of energy deposition culminates to ectopic lipid accumulation in tissues such as muscle, liver, heart and arterial wall. Consequently, there is a severe and early insulin resistance, diabetes mellitus, hepatic steatosis and premature atherosclerotic disease, which may lead to early death^1,2^.

CGL diagnosis can be based on clinical data including acromegaloid features, acanthosis nigricans, reduction of total body fat, muscular hypertrophy and protrusion of the umbilical scar. Also, laboratory data and imaging tests can show other important health information. The identification and knowledge of CGL by the health care professionals is crucial once it is associated with severe and precocious cardiometabolic complications and poor outcome. Although CGL patient phenotype be quite characteristic, the rarity of this disease and its misdiagnosis with common conditions like undernutrition and athletic shape make difficult an early detection of cases, which may significantly improve the prognosis of these patients^3^.

Image processing may be crucial in phenotyping, diagnosis, and even the identification of rare new diseases^5^. Artificial intelligence (AI), mainly through machine learning, provides algorithms capable of learning from data. Images are one of the types of data that AI, namely deep learning, is more fruitful at analyzing^5^. Convolutional neural networks (CNNs) automatically detect patterns of interest in images and have demonstrated image classification performance above the level of humans^4^. Deep learning algorithms have been implemented in medicine and the application into routine clinical practice is feasible^6^, thus it is an interesting strategy to assist the health professional in the diagnosis for the proper management of patients with rare diseases. Therefore, the aim of this study was to identify congenital generalized lipodystrophy phenotype using deep learning.

## Materials and methods

A deep learning approach model was presented as a detailed experiment with evaluation steps undertaken to test the effectiveness. This study was performed in accordance with the Declaration of Helsinki and was approved by the University Hospital Walter Cantídio Ethics Committee, Fortaleza, Ceara, Brazil (no. 5.364.464). All the CGL patients and their families gave formal consent to participate in the study by signing the free informed consent form prior to their inclusion.

### Population and photography database

The dataset consists of two main categories (training and testing) and three subcategories containing photos of patients with individuals with malnutrition, eutrophic individuals with athletic build and CGL patients. These experiments were based on CGL photography database of patients from Ceará, Northeast of Brazil. These patients represent the second largest number of cases of the syndrome in the country and are followed up by a multidisciplinary team of the regional reference center of the Brazilian Group for the Study of Inherited and Acquired Lipodystrophies (BRAZLIPO).

To optimize artificial intelligence training, face and full body images were used, without strict standardization for patient positioning or image acquisition distance. A total of 337 images of individuals of different ages, children and adults were carefully chosen from medical records and internet open access database. In the search for photographic records published on open access platforms, a literature review was carried out. The searches were carried out in the Lilacs, PubMed and Scielo databases. Descriptors and their combinations in Portuguese and English were used with Boolean operators: “Congenital Generalized Lipodystrophy” OR "Berardinelli-Seip Syndrome" AND “physiopathological mechanisms” OR “phonotype” OR “clinical characteristics”; “Malnutrition” AND “physiopathological mechanisms” OR “phonotype” OR “clinical characteristics”.

The clinical history of the 22 patients followed up at the outpatient referral clinic, whose images were included in the analysis, was assessed through medical records.

### Data augmentation

Several data augmentation methods were employed to artificially increase the size and quality of the dataset. This process helps in solving overfitting problems and enhances the model’s generalization ability during training.

In order to carry out the data augmentation process, geometric transformation techniques were used. Some images were rotated and zoomed using angles arbitrarily chosen by the author. In total, eight processes were chosen, six of which consisted of rotating 45°, 90°, 180°, −90°, −50° or −45°. And the other two consist of zooming the image and rotating 18° or 114°. Initially, the database consisted of 80 photos of people without the syndrome and 257 photos of CGL patients. At the end of the data augmentation, we ensured that the number of images between the two groups was balanced and was obtained a total of 896 images.

### Convolutional neural networks model

To build and train the CNN model, it was used Python 3 and some libraries to help, such as Numpy v1.17.4 and Tensorflow v1.15. All experiments were run on a standard PC without a GPU card and a i5-4210u processor.

**Figure 1:**
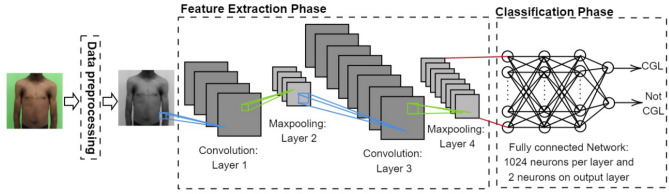
Proposed model for CNN.

Artificial neural network consists of a machine learning model inspired by a neuron, being CNN a class of artificial neural network extremely efficient in processing and analyzing images. The architecture of the proposed CNN model consists of three major phases: pre-processing, feature extraction and the classification (Fig. 1).

The first phase consists of standardizing the images so that the network can treat them all equally, resizing, transforming to grayscale and normalizing the values.

The second phase is responsible for the feature extraction, this phase is to increase the accuracy of the classification models, looking for patterns in a set of pixels. So instead of the network analyzing an image pixel by pixel, this feature extraction is done before, and through some convolution layers together with the pooling layers it is possible to look for some characteristics that the network finds more relevant in the images. For example, this is how we humans would look for eyes, ears, and mouth to determine that the image has a face. In this layer, the network looks for attributes or characteristics that it finds relevant in the images and that can help in classification. It is noteworthy that these features do not always make sense to human eyes, but they are characteristics that can make sense for a computer to identify and differentiate one class from another.

With the features, the third layer is responsible for doing the learning. In this phase several layers of artificial neurons connected to each other try to adjust and identify whether the attributes obtained in the previous phase help to identify the image class. At the end, the prediction of the class is made and compared with the real class. This is possible because in a supervised learning training, which is the case, the network has the information of the real class of each image used in the training.

With the comparison at the end, an analysis of the errors and successes is made to verify if the attributes obtained in the second phase and the adjustments made in the neurons in the third phase were satisfactory or not. If not, the network uses this error analysis to redo the entire process, looking for new attributes and new values for the neurons. This process is repeated until the network learns the best combination of features and values ​​that present satisfactory results.

The hyperparameters used to configure the CNN are shown in Table 1. In this, it is possible to identify that the amounts of convolution and hidden layers are smaller than the amounts of neurons per layers. This motivation was because the computational cost increases exponentially when increasing the number of layers.

**Table 1:** Model hyperparameters.

Number of convolutional layers	2
Filter feature order	[2 × 2]
Number of pooling layers	2
Learning rate	0.001
Number of neurons per layer	1024
Turn-off neurons percentage	0.2
Activation function	ReLu
Maximum number of epochs	600
Number of hidden layer	5

### Validation methods

**Figure 2:**
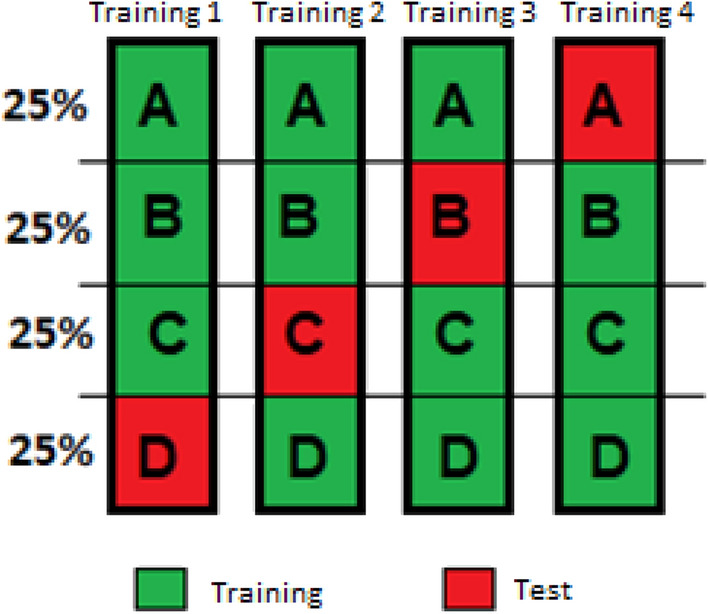
Visual presentation of a fourfold cross validation.

For validation, the dataset was partitioned into four parts, keeping the same proportion of the three subcategories in each part. The fourfold cross-validation technique was applied, using 75% (3 parts) of the data as training and 25% (1 part) as a test. Following the technique, 4 tests were performed, changing the parts that were used as training and testing until each part was used exactly once as validation data (Fig. 2).

## Results

The median age of CGL patients was 16 years, ranging from 1.9 to 42, and 54.5% (12/22) were women. The mean age at CGL diagnosis was 0.6 years, ranging from one month to 41 years of age. All the patients had hypoleptinemia and low HDL-c, 95.5% (21/22) had hypertriglyceridemia, 77.2% (17/22) diabetes, 59% (13/22) hepatic steatosis, 41% (9/22) hypercholesterolemia, and 18.2% 41% (9/22) hypertension. Insulin resistance was evaluated by clinical criteria (acanthosis nigricans) and by calculating the HOMA-IR index, present in respectively 90.9% (10/11) and 81,8% (9/11). HOMA-IR was calculated only in non-insulinized patients. Genetic analysis was performed in all patients. AGPAT2 and BSCL2 gene mutations were identified in 68.1% (15/22) and 31.8% (7/22) of them, respectively.

**Figure 3:**
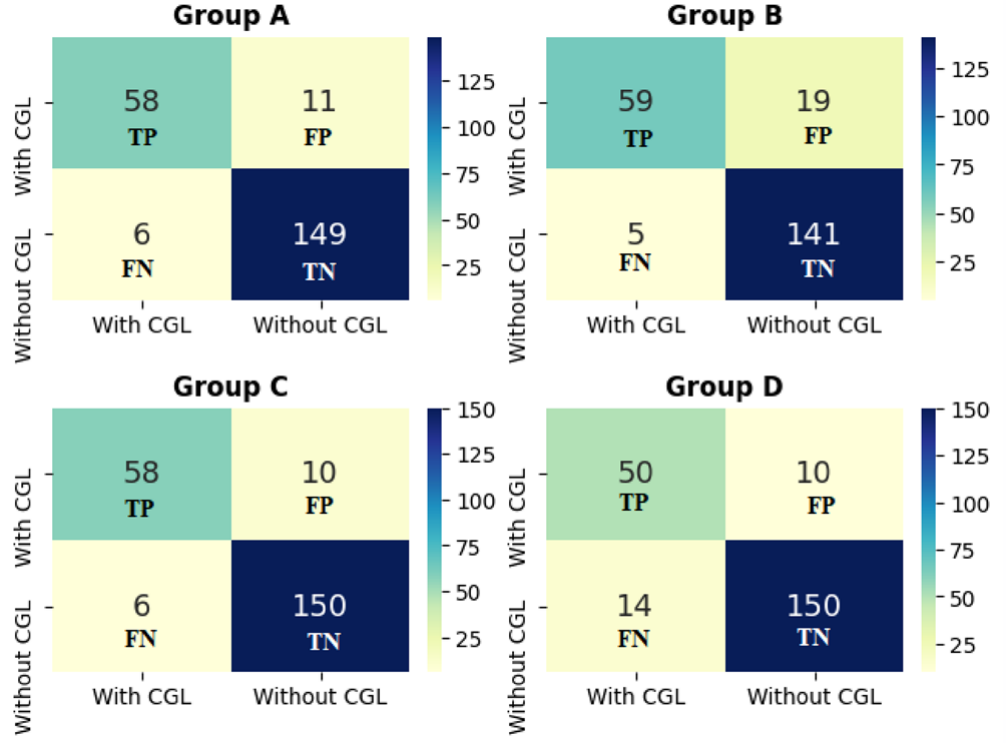
Confusion matrices for each fold. *TP* true positive, *FP* false positive, *FN* false negative and *TN* true negative.

After applying cross-validation, the results associated with each of the four subgroups were composed. Initially, confusion matrices were used to visualize the true positives (TP) and negatives (TN), as well as the false positives (FP) and negatives (FN). With these values, which are shown in Fig. 3, it is possible to raise the capacity that the model generated by the CNN network has in classifying patients with or without CGL. In Fig. 3, in all four subgroups, true positives and negatives have a predominance in relation to false positives and negatives, which is an initial indication showing the good generalization capacity of the chosen model.

With the data obtained from each confusion matrix, other indicators of the model's performance can be measured, such as accuracy, sensitivity, and specificity. The first is indicative of true positive and negative with respect to all cases evaluated. Sensitivity and specificity, which are illustrated respectively, are similar indicatives. The first is the model's ability to correctly predict when the patient does not have CGL, whereas with specificity it is able to correctly predict patients who have CGL.

The mean accuracy, specificity, and sensitivity were obtained with values of [90.85 ± 2.20%], [90.63 ± 3.53%] and [91.41 ± 1.10%], respectively. Regarding the ability to correctly identify patients with CGL, although the test with subset D did not obtain a result above 80%, on sensitivity, it still had a good classification capacity. In terms of specificity, all subsets obtained a rate above 90%, this is a result that coincides with the reality of the data set, since there is a certain level of control in the images of patients without LGC (Table 2). Finally, it is also noted that the computational cost measured in the training stage is also similar for each of the subsets, and this is a relatively low time compared to models with many hidden layers and neurons by layers.

**Table 2:** Results from k-fold CV.

Training	Test	Accuracy (%)	Specificity (%)	Sensitivity (%)	Computational costs (minutes)
A, B, C	D	89.29	93.75	78.13	25.40
A, B, D	C	92.86	93.75	90.63	26.00
A, C, D	B	89.29	88.13	92.19	25.10
B, C, D	A	92.41	93.13	90.63	27.00
	Mean	90.85	90.63	91.41	25.88
	Standard deviation	2.21	3.53	1.10	0.8382

## Discussion

It is estimated that there are more than 7000 rare diseases worldwide, 80% of which are of genetic origin and approximately 75% affect children^7,8^. Although individually rare, collectively, these diseases affect about 350 million people^9,10^. The lack of knowledge about the true frequency of the disease and about specific diagnostic criteria, as well as the lack of official clinical guidelines and the small number of geographically dispersed patients, hinder the clinical diagnosis and recruitment of these patients for clinical research^11,12^.

Images, one of the types of data that artificial intelligence is more productive in analysis, can be used in phenotyping, diagnosis and even in the identification of rare new diseases^13^. This is the first study to present a deep learning model for the analysis of photographic image of CGL patients and identification of phenotypic characteristics.

CGL is a low prevalence condition, affecting 1: 10,000,000 live births, but it is believed that of every four existing cases, only one is reported. Between 300 and 500 patients affected by LGC have already been reported in the medical literature^14^, with a greater concentration of cases in Lebanon, Brazil, Portugal, Scandinavia, as well as in families with African ancestors^15,16^.

The phenotype of the CGL patients is quite characteristic and appear early, in the first years of life, however the rarity of this disease and the lack of knowledge of health professionals make it difficult to identify the clinical manifestations. Many features of the syndrome can be identified through close observation during clinical evaluation. The scarcity of subcutaneous adipose tissue gives patients a characteristic muscular appearance (muscular pseudohypertrophy) with prominence of superficial subcutaneous veins. acromegalic facies, large hands and feet, acanthosis nigricans and umbilical protrusion can also be observed^14^.

Other diseases or disorders in nutritional status may have similar clinical manifestations and physical characteristics. Thus, the development of technologies that involve machine learning can assist clinical evaluation, especially in conditions where remote assistance may be necessary.

LipoDDx^®^ is a free mobile application for the identification of different subtypes of lipodystrophies, which is effective in approximately 80% of cases in this first validation process. This is the first app to allow identification of a set of heterogenous rare diseases, however, a sequence of responses is requested, with no photographic analysis as strategy for identifying diseases, as proposed in the present study^17^. Gurovich et al. (2019) presented a facial analysis framework for genetic syndrome classification called DeepGestalt. This framework leverages deeplearning technology and learns facial representation from a largescale face-recognition dataset, followed by knowledge transfer to the genetic syndrome domain through fine-tuning. The proposed method presents more insights in the applicability of deep learning methods for detection of phenotype in rare disease^18^. Previous research shown an AI expert system that calculates disease probabilities based on patient symptoms that can potentially accelerate rare disease diagnoses^19^.

It is estimated that in Brazil there are approximately 100 CGL patients being followed up in specialized services, however, not all cases are published^20,21^ and access to images is very limited. The relatively small number of patients and the wide range of age represent a limitation and a challenge in machine learning. Nevertheless, our series is one of the largest of the world and the data augmentation technology allowed expanding the number of images and obtaining a satisfactory result.

In conclusion, this study presented for the first time a deep learning model able to identify congenital generalized lipodystrophy phenotype with good accuracy and sensitivity, above 90%, even when using tests with confusing images, with similar characteristics. These deep learning algorithms can be implemented into routine clinical practice, assisting health professionals in the diagnosis of patients with this rare disease. In future work we intend to evaluate a algorithm for automatic detection of a genotype–phenotype correlation. The use of this tool through cell phone applications will facilitate access to this technology, reaching health services in the most remote regions and transforming patient care.

## Data Availability

The datasets used and analyzed during the current study are available from the corresponding author on reasonable request.
